# Transcriptomic and Histological Analysis of Exacerbated Immune Response in Multidrug-Resistant *Pseudomonas aeruginosa* in a Murine Model of Endophthalmitis

**DOI:** 10.3389/fimmu.2021.789023

**Published:** 2022-01-03

**Authors:** Poonam Naik, Suchita Pandey, Milind N. Naik, Dilip Kumar Mishra, Sreedhar Rao Boyenpally, Joveeta Joseph

**Affiliations:** ^1^ Jhaveri Microbiology Centre, Brien Holden Eye Research Centre, L V Prasad Eye Institute, Hyderabad, India; ^2^ Center for Doctoral Studies, Manipal Academy of Higher Education, Karnataka, India; ^3^ Ophthalmic Plastic Surgery & Facial Aesthetics, L V Prasad Eye Institute, Hyderabad, India; ^4^ Ocular Pathology Services, L V Prasad Eye Institute, Hyderabad, India

**Keywords:** endophthalmitis, multidrug resistance, *P. aeruginosa*, host immune response, transcriptome

## Abstract

Multidrug-resistant (MDR) endophthalmitis is a serious threat to the whole spectrum of therapeutic procedures associated with the risk of managing and preventing vision loss. We have earlier shown the interplay of immune mediators in patients with MDR *Pseudomonas aeruginosa* (PA) endophthalmitis leading to worse outcome. Expanding on these findings, a murine model of endophthalmitis was developed to explore the effects of drug resistance on the pathogenesis by analyzing the temporal changes in retinal morphology along with its transcriptomic signatures. Clinical isolates of susceptible (*S-PA*) and multidrug-resistant PA (*MDR-PA*) were injected intravitreally in C57BL/6 mice followed by enucleation at 6 and 24 h time points postinfection. Disease progression and retinal changes were monitored by clinical and histological assessment and transcriptome analysis in a pair-wise manner. Histological assessment of *MDR-PA* eyeball revealed higher disease severity (*p* < 0.05), CD45+ cells (*p* = 0.007), MPO+ cells (*p* = 0.01), GFAP+ (*p* = 0.02), along with higher retinal cell death in mice infected with *MDR-PA* (*p* = 0.008). Temporal transcriptome analysis revealed differential expression of nearly 923 genes at 6 h p.i. and 2,220 genes at 24 h p.i. (FC ≥2, adjusted *p*-value <0.05). Pathway enrichment analysis identified differential regulation of chemokine- and cytokine-mediated, MAPK, and NF-кβ signaling pathways. In conclusion, rapid deterioration of retinal architecture and immune exacerbation was significantly associated with the MDR endophthalmitis, suggesting the need for immunomodulatory agents to strengthen host cell functions and support antibiotics to save the retinal structure from inevitable deterioration and restoration of the vision.

## Introduction

*Pseudomonas aeruginosa*-associated endophthalmitis is often multidrug resistant (1, 2) causing fulminant intraocular infection with higher evisceration and enucleation rate (3–5) and loss of the eye itself. The differences in clinical severity in case of multidrug-resistant *Pseudomonas aeruginosa* (*MDR-PA*) endophthalmitis may lie not only on the toxins produced by the organism but also the magnitude of intraocular inflammation it incites or the extent of immune privilege it undermines. Nevertheless, vision restoration in the case of *MDR-PA*- induced endophthalmitis is low compared with that of drug-susceptible endophthalmitis (2). In a clinical setting, *MDR-Pseudomonas aeruginosa* endophthalmitis is often associated with a poor visual prognosis even with early treatment with second line of latest intravitreal antibiotics to which the isolates are susceptible (2, 6). Similarly, Parchand et al. (7) also reported an overall poor visual outcome in 85.5% of the *MDR-PA* endophthalmitis cases despite the usage of topical steroids. About 37% of patients required evisceration while 30.6% of the eyes progressed to phthisis bulbi, and only 14.5% of eyes had visual acuity better than 20/200 (7). This poor visual outcome despite treatment with second line of antibiotic warrants the need for alternative therapy in *MDR-PA* endophthalmitis. Recent studies have hypothesized that bacterial activation of the host’s immune response also significantly contributes to the inflammation, characterized by the infiltration of cytokines and chemokines (8). The extent and duration of host immune response can influence the outcome of the endophthalmitis, which may either constrain the infection or promote dissemination with subsequent retinal damage. Several studies have characterized the role of altered host immune response involving cytokine regulation in MDR-tuberculosis infection and experimental pneumonia model (9–11) along with dysfunction of the Treg cells (12) and altered activation of cytotoxic CD8+ T cells (13), driving differential metabolic reprogramming (14). The inflammatory response is composed of molecular events followed by multiple signaling pathways which further leads to regulation of JAK/Stat signaling, MAP-kinase signaling, interferon signaling, iNOS signaling, NOD-like receptor signaling, Toll-like receptor signaling, CD40 signaling, STAT3, CD27 signaling, estrogen receptor signaling, glucocorticoid receptor signaling, protein ubiquitination, AMPK, NF-кβ, and apoptosis-related (TNFR) pathways (15). Given the diversity of immune response towards the multidrug-resistant pathogen, it is therefore imperative to understand the immune responses in this immune privileged organ to help reduce infection without substantial damage to the eye.

We have earlier reported an exacerbated immune response of interleukin (IL)-6, IL-1α, IL-8, IL-10, IL-1β, tumor necrosis factor-alpha (TNF-α), and interferon-gamma (IFN-γ) in retinal and microglial cells exposed to MDR *P. aeruginosa* strains (16) as well as correlated this to levels in vitreous of patients with MDR-*P. aeruginosa* endophthalmitis (17), suggesting that *MDR-PA* infections have an excessive inflammatory response which affects restoration of vision. An adjunct approach through the use of immunomodulators that will regulate the expression of specific pathways or mediators, thus reducing ocular inflammation and restraining collateral damage is warranted. Several studies in endophthalmitis have shown the efficacy of immunomodulators in controlling the inflammation (8). A study by Parkunan et al. (2015) reported that *B. cereus*-infected eyes of TLR4−/− mice had significantly less polymorphonuclear leukocyte influx and reduced concentrations of four inflammatory mediators (18). Singh et al. (2021) also reported that intraocular administration of itaconate protects mice by reducing inflammation, bacterial burden, and preserving retinal architecture and visual function (19). Thus, a “fine-tuned optimal” response is necessary to ensure resolution of inflammation. We suggest here that depending on the type of insult and the following inflammatory response, distinct classes of inflammatory pathways can be delineated. However, although it is recognized that antibiotic-resistant strains drive distinct immune response, there remains a large gap in our understanding the significance of host response during the MDR endophthalmitis, which is a common phenomenon in gram-negative infections in our country. To expand on our findings, we performed a comparative transcriptomic and histologic study using two clinical isolates, *MDR-PA* and its susceptible counterpart. Our study provides proof of the need for immunomodulatory strategies targeting the signaling pathways involved in endophthalmitis and can be extrapolated to other multidrug-resistant infections as well.

## Materials and Methods

### Ethics Statement

The experiments described here involved the use of mice and included both male and female C57BL/6 mice (8 weeks of age; Sipra Labs, Hyderabad, India). All procedures were conducted according to guidelines and recommendations of the Guide for the Care and Use of Laboratory Animals by Institutional Animal Ethics Committee (IAEC), Sipra Labs, Hyderabad, India. The study was approved under protocol SLL-PCT-IAEC/20-21/B. The animals were also maintained according to institutional guidelines and the ARVO Statement for the Use of Animals in Ophthalmic and Vision Research.

### Bacterial Culture Preparation

The clinical strains of *MDR-PA* (L-2051/18) and *S-PA* (L-2050/18) were isolated from the vitreous of patients clinically diagnosed with infectious endophthalmitis at our institute. Overnight cultures were grown to logarithmic phase in brain heart infusion broth and serially diluted in saline to approximately 10,000 colony-forming units (CFU)/μl for intravitreal injection, as described below.

### Experimental *P. aeruginosa* Endophthalmitis Model

C57BL/6 mice were anesthetized with a mixture of ketamine and xylazine, and topical anesthetic (0.5% proparacaine HCl) was instilled in each eye before injection. Intravitreal injections were carried out just posterior to the superior limbus, and 1 μl volume was injected directly into the midvitreous, using a surgical microscope (ZEISS Stemi 508 Stereo Microscope, Jena, Germany). The contralateral eyes of all mice were injected in a similar manner with 1 μl of phosphate-buffered saline. All eyes were assessed throughout the course of infection by slit lamp biomicroscopy, while immunohistochemical and histologic analyses, along with quantification of bacterial growth and microarray analysis, were performed after enucleation of the eye at 6 and 24 h postinfection (p.i.).

### Clinical Evaluation

The clinical changes occurring during the induced experimental endophthalmitis were scored independently by an ophthalmologist who was blinded to the type of injection using a hand-held slit-lamp biomicroscope (PSLAIA-11, Appasamy Associates, Chennai, India). Ocular inflammation was scored based on anterior segment inflammation, presence/absence of red reflex, vitreous inflammation, and retinal clarity. Clinical changes were graded on a scale from 0 to 4+ based on the criteria described earlier (20). Photographs of mouse eyes were taken for visualization of progression of disease severity.

### Microbiological Evaluation of Intraocular Bacterial Growth

*MDR-PA*- and *S-PA*-infected eyes were harvested at 6 and 24 h p.i. The eyeballs were placed in 400 µl of sterile phosphate-buffered saline on ice and homogenized (EzLyser Genetix Biotech Asia, New Delhi, India) with 1.0-mm glass beads for 90 s at maximum speed. The homogenates were serially diluted and plated on BHI agar plates. Values represent the means SEM for 3 eyeballs at each time point.

### Histopathological and Immunohistochemistry Analysis

For histopathological scoring of endophthalmitis, eyeballs were enucleated and fixed in 10% neutral buffered formalin, then embedded in paraffin for sectioning at indicated time points. Tissue sections were cut through the pupillary-optic nerve axis. The sections were then stained with hematoxylin and eosin (H&E). Disease severity was assessed by a pathologist who was blinded to the tissue, slide identity, and strain used, using defined grading criteria ranging from 0 (no disease) to 4 (maximum disease) based on the extent of inflammation in the cornea, anterior chamber, vitreous, and integrity of retinal architecture. Eyes that went into panopthalmitis were given a score of 4. Further sections were additionally assessed by gram stain, CD45 (leukocyte marker), MPO (neutrophil marker), and GFAP (astrocyte and muller glia marker) staining and examined using an Olympus light microscope (BX51). Briefly, the procedures included acetone fixation and nonspecific rabbit serum block, along with anti-CD45, anti-MPO, and anti-GFAP antibody (Dako, Agilent, Santa Clara, CA, USA) labeling followed by chromogenic staining using a DAB substrate (Dako, Agilent) and hematoxylin counterstain. Sections of CD45 and MPO were analyzed by counting the total positive cells in the posterior chamber of ten ×400 random microscope fields per case and averaging the number. The GFAP intensity was evaluated based on different force of IHC expression in investigated groups and include: “negative” (−), “weak” (+), “moderate” (++), “strong” (+++), and their variations.

### TUNEL Assay

Terminal deoxynucleotidyl transferase-mediated dUTP nick-end labeling (TUNEL) assay-based protocol (Click-iT^®^ TUNEL Alexa Fluor^®^ 488, Thermo Fisher Scientific, Waltham, MA, USA) was used to assess extent of DNA fragmentation as an indicator of apoptotic death within the entire retinal thickness. DNase I enzyme was used to generate strand breaks in the DNA to provide a positive TUNEL reaction. The coverslips were washed with molecular biology-grade water. Followed by this, reaction mixture was prepared using deionized water, DNase I buffer, and DNase I. A total of 100 µl of the above solution was added to coverslip and was incubated for 30 min at room temperature. After the incubation, the coverslips were then washed once with deionized water and was subjected to TdT reaction. Images were taken using an upright AXIO Imager.M2 Zeiss fluorescence microscope under a ×20/0.8 lens. For each eye, 3–4 serial sections were taken. Random images were also counted by pathologist who was blinded to the tissue, slide identity, and strain used while TUNEL^+^ cell density was calculated as the ratio of TUNEL^+^ cells/mm of the length of the region of interest using an automated ImageJ macro as previously described (21).

### RNA Sample Preparation

Total RNA from mice eye ball (*n* = 3) from each group was isolated by Qiagen RNA extraction followed by DNase I treatment. Quality and quantity of isolated RNA was evaluated by determining RNA integrity number (RIN) using an Agilent 2100 Bioanalyzer (Agilent Technologies, Santa Clara, CA, USA) with nanochip systems. Total RNA with RINs ≥7 was selected for control and infected eyes at each time point for gene expression microarray analysis.

### Gene Expression Profiling: Sample Labeling and Microarray Hybridizations

The mouse tissue gene expression profile was assessed at two time points (6 and 24 h) postinfection with *MDR-PA* and *S-PA* using SurePrint G3 Mouse Gene Expression Microarrays (Agilent, Santa Clara, CA, USA) containing 60,000 probes, allowing for the analysis of >30,000 well-characterized mouse genes and long noncoding RNAs. To identify statistically significant differences in gene expression, the microarray analysis was performed on 3 separate biological replicates at each time point. Briefly, total RNA from each sample was amplified and transcribed into fluorescent cRNA with using Agilent Quick Amp Labelling protocol (version 5.7; Agilent Technologies). RNA samples from *MDR-PA*-infected mice were labeled with Cy5 (red), and Cy3 (green) was used for *S-PA*-infected mice eye. The labeled cRNAs were hybridized onto SurePrint G3 Mouse Gene Expression Microarrays (Agilent, Santa Clara, CA, USA). After hybridization at 65°C for 17 h, subsequent to washing of the slides with washing buffer, the arrays were scanned and analyzed using the Agilent Scanner G2505C (Agilent Technologies, Inc. Agilent Feature Extraction software (version 11.0.1.1; Agilent Technologies, Inc.).

### Microarray Analysis

The probe sets of two dye arrays were annotated with Enterz Identification (ID) using the Bioconductor and the DAVID gene-ID conversation tool. The differential gene expression analysis was performed with limma package in R (v4.0.0.)/Bioconductor (v3.11). After applying background correction within the arrays and loess normalization, a linear model was fitted across the samples of two groups (3 biological replicates each) to estimate the differential expression of each gene. Bonferroni correction was applied to *p*-value, and Benjamini Hochberg correction was used for false discovery rate A gene is said to be expressed if it has an absolute log2 fold change of more than or equal to 1 and an FDR of less than 0.1. The fold change was calculated over 3 biological replicates to reduce false-positive rates.

### Principal Component Analysis and Hierarchical Clustering

In order to study the similarity between the biological replicate of the *MDR-PA*- and *S-PA*-infected groups, we perform principal component analysis on the expression data using R statistical package. The scoring and loading plots for the first two components were created. The gene expression patterns across the two groups were studies by performing hierarchical clustering in R to create heatmaps. These heatmaps also revealed the extent of the separation between the two groups and samples.

### Functional Annotation of Candidate Genes and Pathway Analysis

The differential expression of genes between the two groups were examined for the enrichment of the pathways compiled in Kyoto Encyclopedia of Genes and Genomes (KEGG) database using online DAVID tool. For a given set of DEGs from our analysis, an enrichment analysis was performed using the official gene symbol as an identifier for the species *Mus musculus* to obtain a functional annotation chart consisting of enriched pathways in the KEGG database. Venn diagrams were also created using Venny (1.0) to illustrate overlap between data points in these gene sets.

### Western Blot Analysis

Cell lysate samples were generated using RIPA buffer supplemented with phosphatase and protease inhibitors (50 mM Tris-HCl pH 7.4, 1% (*v*/*v*), 150 mM, NaCl, 0.5% (*w*/*v*) sodium deoxycholate, 1 mM EDTA, 0.1% (*w*/*v*) SDS, 1 mM Na_3_VO_4_, 1 mM PMSF) and protease inhibitor cocktail (Sigma). Protein samples were denatured using sample buffer containing reducing agent (BioRad, hercules, CA, USA) and heated at 70°C for 10 min. Samples were separated on a 10% polyacrylamide gel and subsequently transferred to PVDF membrane (BioRad). The membrane was then blocked in 5% nonfat milk in TBS-T (PBS with 0.1% Tween20) for 1 h at room temperature followed by incubation with primary antibodies of MMP-9 (Abcam, Cambridge, MA, USA), phospho-MAPKAPK2 (CST), NF-кβ-p65 (CST), and β-actin (Santa Cruz, Dallas, TX, USA), overnight at 4°C. The membrane was then washed three times in TBS-T followed by a 1-h room-temperature incubation with IRDye 680RD goat antimouse IgG (LI-COR, Lincoln, NE, USA) secondary antibody. Followed by the incubation, membrane was washed three more times with TBST. Images were captured using Licor Odyssey CLx Imaging System (LI-COR Biotechnology, Lincoln, NE, USA). Abundance was determined by standard densitometry analysis, using ImageJ software (NIH, Bethesda, MD, USA) with β-actin as normalizing protein.

### RT-qPCR Validation

First-strand cDNA was synthesized from equal RNA amounts using the Verso cDNA Synthesis Kit (Thermo Scientific). cDNA was further amplified using primers for mouse caspase-1, caspase-4, caspase-8, NLRP3, and β-actin (internal control) using SYBR Green Master Mix (Thermo scientific).

### Multiplex Immunoassay

Mice cytokine/chemokine magnetic bead panel (MCYTOMAG-70K, Merck Millipore, Darmstadt, Germany) was used for the Milliplex ELISA. The concentrations of 3 human mediators (IL-1β, IL-6, and TNF-α) were measured using a multiplex assay instrument (MAGPIX; Bio-Rad Laboratories, Hercules, CA, USA) according to the manufacturer’s instructions. Standard curves of known concentrations of recombinant human cytokines (R&D Systems, Minneapolis, MN) were used to convert fluorescence units to cytokine concentration (picograms/milliliter). The protein concentration of each sample was determined using BCA method (G-Biosciences, St. Louis, MO, USA).

### Statistical Analysis

An unpaired, two-tailed Student’s *t*-test was used to determine statistical significance for data from the cytokine ELISA and bacterial count. A nonparametric Mann-Whitney *U* test was performed for clinical score, histological score, and TUNEL-positive cells. A *p*-value <0.05 was considered statistically significant. Data are presented as means ± SEM or SD as indicated in the figure legends. Statistical analyses were performed using GraphPad Prism 9 (version 9; GraphPad Software Inc., La Jolla, CA, USA). R scripts were used to construct principal component analysis (PCA) plots, volcano plots, heatmap, and the pathway enrichment plot. All error bars are defined in the figure legends. Statistical methods were used to predetermine sample size.

## Results

### Clinical and Histologic Assessment of Disease Progression in an Experimental Murine Model of Endophthalmitis Infected With MDR-PA and S-PA

**Figure 1:**
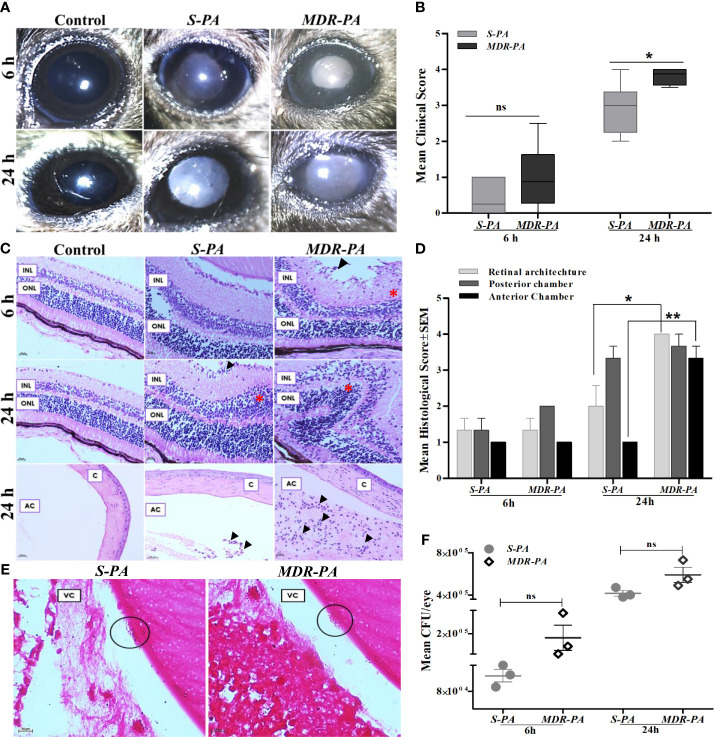
Clinical assessment and histopathological analysis of disease progression and intraocular bacterial growth sequelae following *MDR-PA* and *S-PA* challenge of mice eyes. **(A)** C57BL/6 mouse eyes were injected with 10^4^ CFU of *S-PA* and *MDR-PA* for 6 and 24 h and were clinically scored by an ophthalmologist, who was blinded to the type of infection. **(B)** Data were plotted using box-whisker plots showing median, 25th–75th percentiles, and min–max range (two-tailed Mann-Whitney *U*). Each point shows the average of scores from *n* ≥ 12 in each group. Whole eyes were harvested for histological analysis at 6 and 24 h p.i. Representative H&E-stained sections of the posterior and anterior segments (bottom) of eyes **(C)**. Pathological features indicated retinal folding (asterisk). INL, inner nuclear layer; ONL, outer nuclear layer; C, cornea; AC, anterior chamber. Original magnification, ×400. Severity of disease in the mice eye was assessed by histological scoring in three major categories by a pathologist who was blinded to the type of infection and time point **(D)**. Each point shows the average of scores from *n* = 4 in each group range (two-tailed Mann-Whitney *U*). Values represent mean ± SEM. Gram staining was performed on eyes infected with both S-PA (left) and MDR-PA (right), harvested at 24 h to visualize the intraocular bacteria (black circle). VC, vitreous; **(E)** Original magnification, ×1,000. Quantification of bacteria was done at both 6- and 24-h range (two-tailed Mann-Whitney *U*). Values represent mean ± SEM. Data are representative of at least four similar experiments with 3 mice per group **(F)**. ns, not significant, ^*^ *p* < 0.05, ^**^ *p* < 0.01.

Intravitreal injections of 10^4^ CFU/eye resulted in reproducible clinical endophthalmitis as assessed and graded clinically and histologically with mice having corneal haze, vitreous haze, and intraocular inflammation (**Figure 1A**). Disease severity measured by clinical scoring, showed a time-dependent progression which also correlated with the histopathological grading. Although *MDR-PA* showed early clinical signs of endophthalmitis, the average clinical score of *MDR-PA* did not differ significantly at 6 h from *S-PA* mice (*p* = 0.15); however at 24 h, the difference in mean clinical score was statistically significant (*p* = 0.01) (**Figure 1B**). At 6 h p.i., minimal flare in the anterior chamber was observed with slight diminution in red reflex. Inflammatory symptoms progressed at 24 h p.i. with intense flare, vitreous haze, and a significant decrease in red reflex. Inflammatory symptoms were more severe in *MDR-PA*-infected mice, with haze in the anterior chamber, along with severe iritis and vitreous and retinal clarity was completely obscured by infiltrates. In contrast, control animals had a clear cornea and anterior chamber with no visible signs of inflammation.

Gross histopathological examination of enucleated globes of infected eyes showed inflammation characterized by robust inflammatory infiltrate and disruption of retinal architecture along with retinal folds. The severity of disease was assessed by histological scoring in three major categories: inflammation in posterior and anterior segments and integrity of retinal architecture. Microscopic examinations of the control eyes were consistent with lowest histological scores with no signs of inflammation and intact retinal integrity (**Figure 1C**). At 6 h p.i histological analysis revealed mild inflammation with minimal retinal folds which was higher in *MDR-PA*-infected mice eye, though the difference in mean histological scores was not statistically significant (*p* > 0.05). On the contrary, sections from the eye of *MDR-PA* recipients at 24 h displayed showed greater accumulation of inflammatory cells, with higher retinal folds and indistinguishable retinal layers (*p* = 0.01). The anterior segment of the *MDR-PA*-infected mice eye showed significantly higher inflammatory cells (*p* = 0.008) and higher retinal layer disruption (**Figure 1D**). Additionally, severe periorbital and surrounding tissues indicated panopthalmitis. Taken together, these observations demonstrate that the disease severity was higher in *MDR-PA*-infected mice, which worsened with time as compared with the *S-PA*-induced endophthalmitis.

### Microbiological Evaluation of Intracellular Growth

Next, we evaluated the intraocular bacterial growth as the ability to replicate and survive within host cells after infection, as critical determinants of disease pathogenesis. *MDR-PA*-infected mice eye had higher number of colony forming units (CFU) at both 6 and 24 h, though this difference was not statistically significant (*p* = 0.16 at 6 h, *p* = 0.08 at 24 h) (**Figure 1F**). Grossly, however, gram staining of the eye section of the mice confirmed the presence of rod-shaped bacilli in the vitreous cavity only at 24 h p.i. (**Figure 1E**), while it was not visible at 6 h p.i.

### MDR-PA Induces Exacerbated Inflammation (CD45+, MPO+) and Elevated Retinal Stress (GFAP)

**Figure 2:**
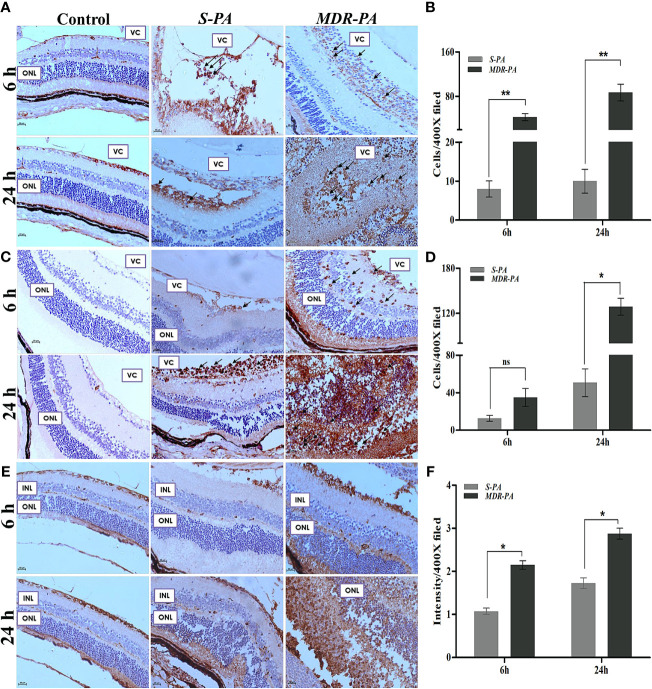
CD-45, MPO, GFAP, and retinal cell death against *MDR-PA* and *S-PA* infections. C57BL/6 mice eyes were injected with 10^4^ CFU of *S-PA* and *MDR-PA*. Infected eyes along with uninfected control were collected for histological assessment. The tissue sections were stained, and positive cells were counted CD-45 **(A)**, MPO **(C)**, and GFAP **(E)** staining with approximately 4 mice per time point. Original magnification, ×400. Arrowheads indicate inflammatory cell infiltration. The bar graph represents the average of CD45-positive cells **(B)** and MPO-positive cells **(D)** observed in tissue sections. Values represent the mean ± SEM. Quantification of relative GFAP intensity per high-power field **(F)**. Data were analyzed with two-tailed Mann-Whitney *U* test. At least 10 randomly selected fields per tissue section were analyzed by an observer blinded to genotype. The graphs show mean ± SEM. Significance was calculated using Mann-Whitney *U* test. Data were combined from 3 independent experiments where *n* = 3 in each group; ns, not significant, ^*^ *p* < 0.05, ^**^ *p* < 0.01.

To quantify the inflammatory cell infiltration, the number of the CD45+ and MPO+ cells were counted in the posterior segment, then the mean value was calculated. We observed a significant increase in the number of CD45+ and MPO+ cells in *MDR-PA*-infected mice eye. Furthermore, the number of CD45+ cells were significantly lower in *S-PA*-infected mice eye at both the 6 h p.i. (*p* = 0.007) and 24 h p.i(p= 0.007) (**Figures 2A, B**). The CD45+ cells in *MDR-PA*-infected mice eye displayed progressive increase over time, whereas in the case of *S-PA*-infected mice eye, CD45+ cells showed no appreciable change over time. Higher MPO+ cells were found in *MDR-PA*-infected mice eye only at 24 h p.i (*p* = 0.01) (**Figures 2C, D**). To assess the extent of differential retinal glial (astrocytes and muller glia) stress following *MDR-PA* and *S-PA* infection, we used GFAP at both the time points. *MDR-PA*-infected mice retinas showed significant increase in immunoreactivity for GFAP, suggesting an increased retinal stress. As seen in **Figures 2E, F**, the intensity of GFAP was robustly increased in *MDR-PA*-infected mice eye at both 6 and 24 h compared with the susceptible (*p* = 0.02, *p* = 0.03, respectively; *n* = 4).

### Infection With MDR Induces More Retinal Cell Death

**Figure 3:**
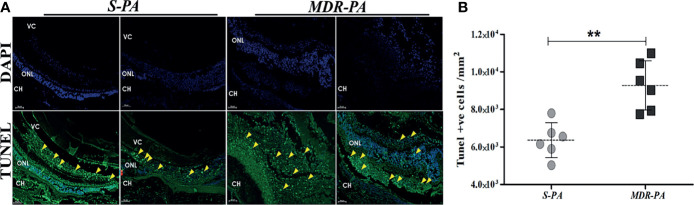
Increased retinal cell death in *MDR-PA* infection. C57BL/6 mice eyes were injected with 10^4^ CFU of *S-PA* and *MDR-PA*. Infected eyes along with uninfected control were collected for histological assessment. Apoptosis within the retina was determined by a TUNEL-based assay and counterstaining was performed with 4′-6-diamino-2-phenylindole (DAPI) after 24 h post-infection with *MDR-PA* and *S-PA* **(A)**. TUNEL (green) and DAPI (blue); scale bar: 50 μm. **(B)** Graph indicating the number of TUNEL-positive cells in the sections of *S-PA*- and *MDR-PA*-infected mice eyeballs. Significance was calculated using Mann-Whitney *U* test. Data were combined from 3 independent experiments where *n* = 3 in each group; ^**^ *p* < 0.01.

To correlate the retinal stress with retinal cell death, TUNEL assay was carried out at aforementioned time points. Early studies have shown that increased retinal cell death is associated with declined visual function in bacterial endophthalmitis. In the present study, *MDR-PA* induced robust cell death as compared with the *S-PA* infections, as seen in **Figure 3A**. Surprisingly, both *S-PA* and *MDR-PA* infections did not lead to retinal cell death in any layer of the retina at 6 h p.i. At 24 h, the retinal cell layers of MDR-PA-infected mice were indistinguishable followed by higher number of TUNEL+ cells in *MDR-PA*-infected group (9,285.43 ± 535.04) than that in the *S-PA*-infected group (6,363.16 ± 377.98, *p* = 0.008) (**Figure 3B**). Most apoptosis was observed in the inner nuclear layer and outer nuclear layer; however, further immunocytochemical analysis was not done.

### A Cluster of Differentially Regulated Genes in Mice by MDR-PA Endophthalmitis

**Figure 4:**
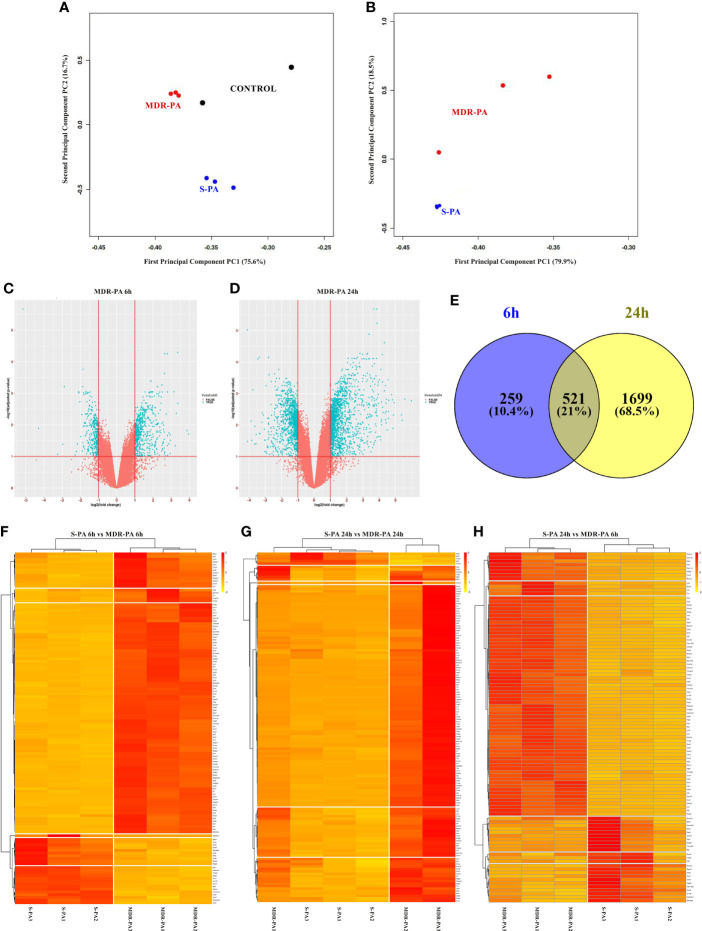
Host transcription profiling identifies a core of host genes differentially upregulated and downregulated by *MDR-PA*. C57BL/6 mice eyes infected with *S-PA* and *MDR-PA* for 6 and 24 h were subjected to RNA extraction, amplification, and labeling and were used to hybridize Agilent microarrays. PCA and volcano plot showing the DEGs in two groups with log2 FC ≥2 (*p* < 0.05) as the threshold. The black dot represents control **(A)**, blue dots represent the three biological replicates of the *S-PA*-infected mice eye ball, and the red dots indicate the biological replicates of the *MDR-PA*-infected mice **(B)**. In the volcano plot, blue dots represent significantly upregulated genes (left) and significantly downregulated genes (right), while the red dots represent the genes which were below threshold. The volcano plot at 6 h **(C)** showed relatively less DEGs compared with the 24 h **(D)**. Venn diagram represents the differentially expressed genes at only at 6 h (blue) and 24 h (yellow) and common (grey) for both **(E)**. Heatmap of the expression of genes differentially expressed (adjusted *p*-value <0.05, fold difference ≥2, FDR ≤0.1) in *S-PA*- and *MDR-PA*-infected mice eyeballs both at 6 h **(F)**, 24 h **(G)**, and S24 vs. R6 **(H)**. Normalized expression levels, arranged by unsupervised hierarchical clustering, reflect overexpression (red) or underexpression (yellow) of genes (columns) for the biological replicates (rows).

In this study, we sought to explore the possibility of finding host genes collectively de-regulated by the *MDR-PA* strain. To this end, microarray was performed on RNA isolated from the mice eye infected with *MDR-PA* and *S-PA* over 6 and 24 h postinfection. The PCA for the 6 h time point indicated a strong correlation between biological replicates and highlights a rapid divergence in transcriptional profile between *S-PA*-infected group and *MDR-PA*-infected group (**Figure 4A**). At 24 h time, one of the biological replicates (**Figure 4B**) was clustered near to the *S-PA* group, while three of the biological replicates from the *S-PA* group clustered together similar to the pattern displayed at 6 h. Despite the differences, the *MDR-PA* group still managed to cluster differently from the *S-PA*-infected group. The analysis revealed distinctive clusters of samples consistent with the biological groups on the basis of the transcriptional profiles. Moreover, a volcano plot was constructed to visualize the DEGs between two groups at the two time points (**Figures 4C, D**). When compared with *S-PA*-infected mice eyes, a total of 1,088 genes at 6 h and 2,900 genes at 24 h were found to be significantly differentially expressed in *MDR-PA*-infected group (FC ≥2, *p* < 0.05). As this variance in number of DEGs has the potential to bias downstream comparisons, we further adjusted the *p-*value to reduce the false-discovery rate to take forward for comparative functional enrichment analysis. With an adjusted *p*-value, we found that a total of 923 genes at 6 h and 2,220 genes at 24h were significantly differentially regulated. Additionally, we also investigated the genes which were only expressed at 6 and 24 h along with the genes which showed temporal expression. In total, 259 genes were found to be expressed exclusively at 6 h while 1,699 genes were expressed at 24 h; 521 genes were found to be expressed at both 6 and 24 h (**Figure 4E**).

To gain insight into the functional relevance of the DEGs, DAVID was used. Some of the DEGs involved in immune regulation were further represented by a heatmap, where not only the number of upregulated genes but also the similarity patterns between the three biological replicates were particularly striking. This suggests that *MDR-PA* infection initially triggers an increased immune response, manifesting as an early qualitative and quantitative changes in gene transcription, which grows rapidly and spreads exponentially over time compared with *S-PA*-infected mice eye. For instance, at 6 h (**Figure 4F**) genes like mitogen-activated protein kinase 1 (Map4k1 (fold change (fc), 2.3), C-type lectin domain family 7 member A (Clec7a, fc = 3.6), Mast cell-expressed membrane protein 1 (Mcemp1, fc = 5.5), macrophage-expressed gene 1 protein (Mpeg1, fc = 3.4), and B-cell leukemia/lymphoma 2-related protein A1d (Bcl2a1d, fc = 3.3) were differentially upregulated compared with the *S-PA*-infected mice group. In contrast, the downregulated genes involved calmodulin-1 (Calm1, fc = 2.2), Fas apoptotic inhibitory molecule 1 (Faim, fc = 2.1), Fas apoptotic inhibitory molecule 2 (Faim 2, fc = 2), G-protein coupled receptor-associated sorting protein 2 (Gprasp2, fc = 2.2), and regulator of G protein signaling (Rgs 7, fc = 2).

At 24 h (**Figure 4G**), the top upregulated genes compared with the *S-PA*-infected group involved lipocalin (LCN-2, fc = 3.5), interferon, alpha-inducible protein 27-like protein 2A (Ifi27l2a, fc = 2.2) which is suggested to be a novel regulator of neuroinflammation in microglia, LPS-induced TN factor (Litaf, fc = 2.4), CD-14 (fc = 14.6) which is a marker for monocytes along with TLR 2 (fc = 2.7), TLR 4 (fc = 5.1), TNF receptor-associated factor 1 (TRAF1, fc = 20.8) whose expression is largely limited to activated immune cells, including myeloid and lymphoid cells. TRAF1 is present at minimal levels in resting lymphocytes and monocytes, and its expression is increased upon activation through the nuclear factor kappa-light-chain enhancer of activated B-cell (NF-κB) pathway. Chemokines like C–C motif chemokine ligand (Ccl)3 (fc = 14.9), Ccl4 (fc = 42.2), Ccl6 (fc = 16.6), Ccl7 (fc = 2.23), and Ccl9 (fc = 6.91) were found to be upregulated. Few of these molecules are previously implicated in endophthalmitis pathogenesis. GFAP was also found to be upregulated at 24 h, which was also observed in the histopathological section. At the other extreme Fas apoptotic inhibitory molecule 1 (Faim 1, fc = 2.9), regulator of G-protein signaling 9 and 7 (fc = 2.73, 2 respectively), ubiquitin-specific peptidase 33 (Usp33, fc = 2.41), and ubiquitin carboxyl-terminal hydrolase Usp2 (fc = 3.81) were among the genes whose expression was downregulated. Reduced USP33 has been shown to decrease the stability of a suppressor molecule of major proinflammatory gene expression pathways of TNF-α, NF-κB, and IFN-β (22). This indicates that regulatory molecules related to apoptotic pathway, GPCR pathway, and suppressors of major proinflammatory pathways are possibly involved in the pathogenesis of drug-resistant endophthalmitis. The extent of differential expression of several genes further intrigued us to construct another heatmap to compare the expression level at 6 h post-*MDR-PA* infection with 24 h post-*S-PA* infection (**Figure 4H**). We observed that even at a 24-h time point, the amplitude of host immune response induced by *S-PA* was secondary to the immune response prompted by *MDR-PA*.

### Cytokine Storm in MDR-PA Endophthalmitis and Role of Complement Mediators

Several studies have addressed the role of cytokines and chemokines (inflammatory mediators) in bacterial endophthalmitis. Because of their importance, we focused on the differentially expressed cytokines and chemokines in *MDR-PA*-infected mice eye at both study time points. At the early time point (6 h), cytokines like in interleukin (IL)-1β (fc = 5.9 ± 0.4), IL-20rb (fc = 2.3 ± 0.12), IL-1rn (fc = 3.9 ± 0.19), IL-10ra (fc = 7.15 ± 0.16), IL-2rb (fc = 5.65 ± 0.11), chemokine (C-X-C motif) ligand (CXCL)-9 (fc = 2.14 ± 0.2), CXCL-5 (fc = 4.6 ± 0.27), C-X-C chemokine receptor (CXCR)-2 (fc = 3.2 ± 0.10), CXCR-3 (fc = 2.3 ± 0.11), CXCR-4 (fc = 2.6 ± 0.12), CC chemokine ligand (CCL)-9 (fc = 2.4 ± 0.04), CXCL-10 (fc = 2.2 ± 0.3), and CXCR-6 (fc = 2.5 ± 0.07) were found to be upregulated. Similarly, inflammatory mediators like CXCL-1 (fc = 3.2 ± 0.01) and CCL-17 (fc = 2.1 ± 0.05) were found to be downregulated. However, during the late hour (24 h) time point, a whole range of inflammatory mediators were found to be upregulated. **Table 1** enlists all the inflammatory mediators, associated proteins, and few regulatory molecules along with its average expression in the three biological replicates that were affected in this cytokine storm. In addition, we observed that components of the complement system were also significantly overexpressed at 24 h p.i. Those include the following, Cfb (fc = 3.4), C5ar1 (fc = 10.2), C1qa (fc = 2.1), C1qb (fc = 2.1), C1qc (fc = 2.4), C3 (fc = 8.1), C4b (fc = 2.7), while C1ql3, which stimulates glucose uptake in various cells, was found to be comparatively downregulated by 3.2-fold compared with the *S-PA*-infected group. Altogether, this suggests an explosive immune response at the later time point, which is likely to be responsible for collateral retinal damage and a good percentage of vision loss.

**Table 1:** Differentially upregulated cytokines and chemokines associated with immune responses during *MDR-PA* infection post-24 h infection.

Inflammatory mediators	Average expression across the samples	Fold change value of *MDR-PA*- compared with *S-PA-*infected mice eye	± Error	Adjusted *p-*value
IL-11	6.57	2.68	0.07	0.0147
IL-15	5.94	2.54	0.02	0.0019
IL-1β	11.09	38.66	7.05	0.0098
IL-1receptor accessory protein	7.59	5.30	0.03	0.0005
IL-4ra	7.49	4.12	1.08	0.0012
IL-7 receptor	5.53	2.41	0.39	0.0015
IL-1 receptor 2	8.53	14.02	2.29	0.0046
IL-10receptor-alpha	5.31	14.6	3.47	0.0016
IL-10receptor-beta	8.86	3.42	0.73	0.0008
IL-6	8.04	2.6	0.33	0.0147
TNF-α	7.13	8	2.6	0.0006
IL-24	8.19	2.7	0.02	0.0055
IL-16	5.72	4.22	1.3	0.0047
IL-1α	6.27	8.92	3.12	0.0162
IL-6 receptor-alpha	8.21	3.65	1.07	0.0019
IL-23 receptor alpha	5.58	7.77	3.06	0.0514
IL-18 binding protein	6.75	2.96	0.35	0.0008
CCL-6	10.14	16.66	4.25	0.0054
CCL-24	5.89	4.47	1.48	0.0021
CCL-12	6.66	2.6	0.13	0.0026
CCL-3	3.9	14.92	4.75	0.0013
CCL-9	8.14	6.91	1.88	0.0002
CCL-4	10.64	42.2	6.10	0.0071
CCL-7	8.78	2.23	0.37	0.0220
CCL-19	6.69	2.60	0.38	0.0011
CXCL-2	8.09	12.1	4.06	0.0189
CXCL-5	10.46	8.52	2.4	0.0005
CXCL-12	6.62	2.1	0.29	0.0127
CXCL-16	7.04	3.2	1.3	0.0045
CXCL-13	5.37	2.09	0.08	0.0036
CXCL-14	7	2.26	0.55	0.0341
CXCL-9	6.72	3.14	0.54	0.0006
Chemokine like factor	6.16	2.18	0.31	0.0029
CXCR-2	7.52	9.81	2.34	0.0011
CXCR-4	5.66	2.86	0.12	0.0022

### Validation of Inflammatory Mediators by ELISA and Inflammasome Activation by qRT-PCR

**Figure 5:**
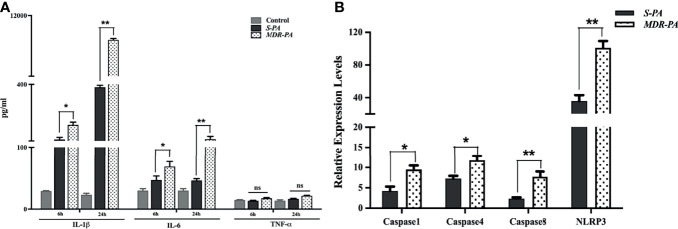
Validation of regulatory molecules using ELISA and qRT-PCR. Multiplex ELISA was performed to check the time-based differential expression of IL-1β, IL-6, and TNF-α **(A)**. All values are averages ± SEM from triplicate samples of 3 individual mice from each condition. mRNA expression of selected caspases was measured by quantitative real-time PCR in *MDR-PA*- and *S-PA*-infected mice at 24 h time point. β-Actin was used as an internal control. All values are averages ± SEM from triplicate samples of 3 individual mice from each condition **(B)**. Statistical analysis performed using Student’s *t*-test **(A, B)** for the comparison of *S-PA*-infected mice vs. *MDR-PA*-infected mice. ns, not significant, *p < 0.05, **p < 0.01.

To validate the role of identified immune mediators that were overexpressed in the pathogenesis of *MDR-PA* endophthalmitis, we analyzed the level of IL-6 and TNF-α by multiplex ELISA. In accordance with microarray, IL-1β in *MDR-PA*-infected eyes was observed to be expressed higher than the *S-PA*-infected ones both at 6 h (192.75 ± 13.69 vs. 117 ± 11.23; *p* = 0.01) and 24 h (10,395.84 ± 86.67 vs. 385.33 ± 10.45; *p* = 0.007). While IL-6 was also observed to be higher at both 6 h (68.43 ± 8.27 vs. 46.74 ± 6.43; *p* = 0.03) and 24 h (119.4 ± 14.01 vs. 46.01 ± 2.93, *p* = 0.009), we could not validate the differential expression of TNF-α by ELISA (**Figure 5A**). Additionally, genes encoding pro- and antiapoptotic/pyroptosis molecules were found to be differentially regulated in *MDR-PA*-induced endophthalmitis and included upregulation of caspase-1, caspase-4, and caspase-8. NOD-, LRR-, and pyrin domain-containing protein 3 (NLRP3) which is known to be a critical component of the innate immune system that mediates caspase-1 activation and the secretion of proinflammatory cytokines was also found to be upregulated. In addition, caspase recruitment domain6 (CARD6), which was observed to be involved in assembly of signal complexes leading to activation of caspase family proteases and induction of NF-кβ was also differentially upregulated. Furthermore, BCL2L15 (B-cell lymphoma 2) which is an amplifier of the apoptotic signal (23) was also differentially expressed in *MDR-PA* induced endophthalmitis. Another important mediator of caspase-8 activation, known as FADD was found to be upregulated. TRAF6 which is believed to be triggered by LPS and interacts with caspase-4 was also upregulated. The interaction of these two molecules leads to NF-кβ-dependent transcriptional upregulation and secretion of cytokines and chemokines. On the contrary, Faim2, which is known as an antiapoptosis gene that directly interacts with Fas receptor and inhibits apoptosis by interfering with caspase 8 was found to be downregulated, along with Fas apoptosis inhibitory molecule (FAIM) which is an antiapoptotic protein. We speculate that the aberrant expression of IL-1β is due to pleiotropic functions of caspases mediated by NLRP3 and is involved in the disease pathogenesis. The above-mentioned observations prompted us to validate the expression of the caspases and NLRP3. To this end, our RT-PCR results were in agreement with the microarray data and we observed differential expression of caspase-1, caspase-4, and caspase-8 and NLRP3 in *MDR-PA* induced infection (**Figure 5B**).

### Differential Expression of MMPs Upon MDR-PA Challenge of Mice Eye

**Figure 6:**
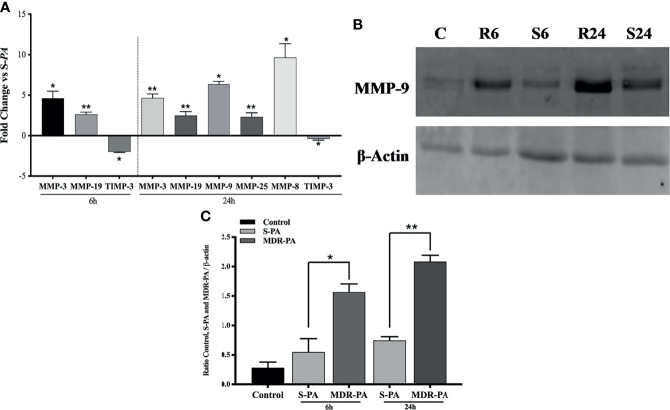
Expression of MMPs. Differential expression of MMPs were plotted to show the time-based upregulation and downregulation **(A)**. The expression of MMP-9 was confirmed by Western blot analysis **(B)**. Equal amount of protein was loaded into the well which was confirmed by β-actin. The intensities of the bands were quantitated by densitometric analysis and presented as a ratio to β-actin **(C)**. ^∗^ *p* < 0.0.05; ^∗∗^ *p* < 0.01; ns, not significant (Mann-Whitney *U* test). C, Control; S6, *S-PA* 6 h; R6, MDR 6 h; S24, *S-PA* 24 h; R24, *MDR-PA* 24 h.

We next explored the expressional variation of matrix metalloproteinases (MMPs) in the *MDR-PA*-infected mice eyeballs which were found to be higher compared with *S-PA*-infected mice eyeballs. These included upregulation of MMP-25, MMP-8, MMP-3, MMP-9, and MMP-19, while the inhibitory molecules like TIMP-2 and TIMP-3 were found to be downregulated at 24 h p.i. Interestingly, at the early time point of 6 h p.i., only MMP-3 and MMP-19 were upregulated while MMP-13 and TIMP-3 were downregulated (**Figure 6A**). Collectively, these findings highlight elevated expression of these peptidases that may contribute to the pathogenesis of *MDR-PA* endophthalmitis by ECM remodeling. Western blot analysis of MMP-9 confirmed its overexpression in *MDR-PA*, while comparatively, lower level of MMP-9 was observed in *S-PA*-infected mice eye at both the time points. These data suggested that the expression of MMP-9 increases over the course of infection caused by *P. aeruginosa* but is significantly higher in the MDR strains (**Figure 6B**). The Western blot band intensities were measured with ImageJ software. MMP-9 protein levels were normalized to respective β-actin levels and were plotted. The difference in band intensity was calculated between *S-PA* vs. *MDR-PA* at both 6 and 24 h (**Figure 6C**) and was found to be higher in *MDR-PA*-infected eyes.

### Role of Signaling Pathways in MDR-PA Endophthalmitis

**Figure 7:**
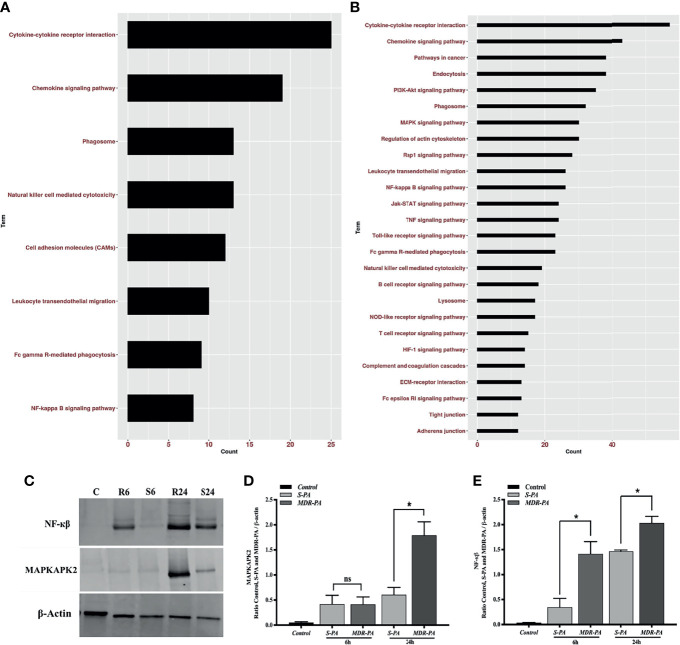
Pathway enrichment analysis of differentially upregulated host genes induced by *MDR-PA* and validation of NF-kB and MAPK at protein level. For the pathway enrichment analysis, genes which were differentially expressed with adjusted *p*-value <0.05, fold difference ≥2, and FDR ≤0.1 were involved. Differentially upregulated pathways at 6 h **(A)** and 24 h **(B)**. For each KEGG pathway, the bar shows the fold enrichment of the pathway. The *x*-axis shows the number of genes, and the *y*-axis shows the KEGG pathway terms. Tissue lysates from *MDR-PA* and *S-PA*-infected eyes were obtained at both the time points and employed for protein expression by immunoblot analysis using specific antibodies to phospho-NF-кβ and MAPKAP2 **(C)**. The intensities of the bands for MAPKAPK2 **(D)** and NF-кβ **(E)** and were quantitated by densitometric analysis and presented as ratio to β-actin. C, Control; S6, *S-PA* 6 h; R6, MDR 6 h; S24, *S-PA* 24 h; R24, *MDR-PA* 24 h. ns, not significant, *p < 0.05.

While the top DEGs provided some interesting insights, it is important to take a systemic approach to the data. Therefore, overrepresented pathways were discerned among DE genes using KEGG pathway, in which all genes were mapped to the reference canonical pathways of KEGG. When the core upregulated genes were sorted into gene ontology categories, the cytokine- and chemokine-mediated signaling pathway emerged as the biological processes with the most significant changes in gene expression at 6 and at 24 h. Accordingly, at 6 h, cytokine-cytokine receptor interaction, chemokine signaling pathway followed by phagosome as well as NF-кβ pathways were the pathways to which upregulated genes could be assigned (**Figure 7A**). Accordingly, several genes that mediate activation of the abovementioned pathways at 6 h are listed in **Supplementary Table 1**. Genes known to have a role in G-Protein-coupled receptor pathway were found to be negatively regulated in *MDR-PA* endophthalmitis and included Rgs7 and Gprasp2. At 24 h p.i., most of the genes upregulated by *MDR-PA* were found to be involved in cytokine-cytokine receptor signaling, chemokine signaling, MAPK signaling, NF-кβ, Toll-like receptor signaling, NOD-like receptor pathway, TNF signaling, and complement system (**Figure 7B**). Several genes that mediate activation of the abovementioned pathways at 24 h are listed in **Supplementary Table 2**. These responses are necessary to limit pathogen infections, but at the same time it needs to be optimally regulated to avoid immune-mediated host damage. When we analyzed the downregulated genes, the pathways involved included Rap1, cAMP, Ras, cGMP-PKG, inflammatory mediator regulation of TRP channels, phototransduction, and phosphatidylinositol signaling system.

Out of all the enriched pathways by the differentially upregulated genes, we further validated NF-кβ and an important regulatory molecule of MAPK pathway MAPKAPK2(MK2) by Western blotting using phosphorylated antibodies (**Figure 7C**). While NF-кβ was expressed differentially at both the time points, MK2 was differentially expressed only at 24 h. The Western blot band intensities were measured with ImageJ software. MAPKAPK2 (**Figure 7D**) and NF-кβ (**Figure 7E**) protein levels were normalized to respective β-actin levels and were plotted. The difference in band intensity was calculated between *S-PA* vs. *MDR-PA* at both 6 and 24 h. These results indicated that Western blot data were consistent with the microarray analysis. Together, these results suggest that NF-кβ and MAPK may be facilitating the increased inflammation.

## Discussion

Our study presents the first in-depth analysis of differential immune pathogenesis in endophthalmitis caused by multidrug-resistant *Pseudomonas aeruginosa* compared with its antibiotic susceptible strain. While most studies focus almost exclusively on the virulence of the pathogen and the mode of action of the antibiotic, without explicitly considering the host immune response, few studies do suggest that the severity and outcome of an infectious disease is influenced by host-pathogen interactions (24). Analysis of human vitreous samples collected from different patients with endophthalmitis corroborated our *in vitro* observations of differential immune response (16, 17). To further confirm this topological cluster of differential expression, we created an *in vivo* murine model of endophthalmitis for a comprehensive high-throughput gene expression profiling. In the present study, both histopathological and transcriptome analysis confirmed excessive expression of numerous immune mediators in *MDR-PA*-infected male and female mice eyes leading to elevated retinal stress and retinal cell death and damage at a dose of 10^4^ CFU/µl. While the strains used were clinical isolates, we conducted a preliminary study (data not shown) on various doses (1,000, 5,000, 10,000, and 20,000 CFU/µl) to understand the efficacy of our disease model. Our goal was to define a minimal dose infecting all animals in an experiment (e.g., AID_99_) following intravitreal injection to reproducibly mimic the clinical severity seen in patients within 24 h. All scoring and characterization were done by an ophthalmologist, and we observed that while 20,000 CFU resulted in complete destruction of the mice eye/panopthalmitis by 8 h, 5,000 CFU took more than 48 h to establish a visible infection. With a dose of 10,000 CFU, progressive infection was observed which could be graded over a period of 24 h. Additionally, few other studies on endophthalmitis have used similar dose for other organisms as well (25) which validated our dose to be clinically relevant. In addition, although Singh et al. (26) have reported that there was no significant difference in disease severity and innate responses in male versus female mice in endophthalmitis, several other studies have reported that males and females differ in their immunological responses to foreign and self-antigens and show distinctions in innate and adaptive immune responses (27). Hence, sex should continue to be considered a biological variable to avoid any gender biasness and to drive important discoveries in both basic and clinically relevant research. However, our transcriptomic data had only three biological replicates, and we could not analyze the difference in inflammatory signature molecules based on sex variable, though this particular analysis would have added further insight on gender-based signature inflammatory molecules. Comparing the entire transcriptome of *MDR-PA*-infected mice with *S-PA*-infected mice, we identified genes that are differentially expressed during pathogenesis thereby contributing to severe ocular damage that is associated with clinical MDR infections. One of the important goals of understanding the extent of regulation of gene expression and the biological pathways is to unmask previously unidentified or differentially regulated targets to control excessive inflammation and prevent irreversible bystander damage to the retina. Pathway analysis also concurred that the majority of the gene response in our study overlap with genes associated with inflammatory processes in the public database. The cytokine associations uncovered were however expected based on previous studies (16, 17). Our previous studies also found similar overexpression of these IL-1β, IL-6, IL-10, and TNF-α cytokines in human microglial cells, retinal pigment epithelial cells, and human vitreous samples. Interestingly, IL-1α showed a differential expression in both *in vitro* and *in vivo* mice model infected with *MDR-PA*, but the same could not be corroborated in the human vitreous samples (16, 17). This altercation might be due to the low human samples included in the study. Apart from the involvement of a broad range of cytokines, our transcriptomics data revealed the involvement of a large number of chemokines involved in causing a dysregulated immune response, which included CXCL (2, 5, 9, 12–14, 16), CCL (3, 4, 6, 7, 9, 12, 19, 24), and CCR (2, 5). While several studies have shown the affiliation of these chemokines with inflammation (28), not much has been described on its association with MDR *P. aeruginosa* infection. A study by Coburn et al. (29) has reported the upregulation of CXCL-2, CCL-2, and CCL-3 in *Bacillus endophthalmitis*, leading to extensive retinal damage. They further suggested that CCL3 recruits and activates polymorphonuclear leukocytes through its interactions with CCR5 and their blockage might reduce neutrophil infiltration into the vitreous and thereby decrease damage to the retina. While GFAP is an astrocyte marker, Wu et al. (30) reported that GFAP is an established indicator of retinal stress in both retinal astrocytes and Müller cells (22, 30), while some other studies (31) also report that increased GFAP levels in Müller glia play a role in the extensive structural changes resulting in Müller cell hypertrophy and glial scar formation. In our study, we observed a marked upregulation of GFAP in our microarray data which also corroborated with the histopathological analysis.

Additionally, in the *MDR-PA*-infected mice, we observed a differential upregulation of several caspases along with NLRP3, which is an important component in the formation of the inflammasome complex. Studies have shown that toxins secreted by *P. aeruginosa* (exoT, exoS, and exoU) can activate the NLRP3 inflammasome (32), leading to tissue destruction. Inflammasomes are known to contribute to the pathology of a variety of autoimmune conditions, inflammatory diseases, and cancers (33, 34). While none of the inflammasomes found in our study had a role in protection from the induced cytokine storm in ocular tissues, NLRP3 inflammasome has been shown to have a protective role during infection (35). It has also been suggested that during infection, assembly of the inflammasome *via* IL-1β attracts inflammatory mediators (36–38) and in the process of controlling the pathologic effect, it in turn causes a collateral damage to the host tissue resulting in partial or permanent blindness (35). This process, including the release of cytokines and activation of caspase-1, can lead to pyroptosis (39) which is an inflammatory form of programmed cell death. Upregulation of IL-1β early during infection might indicate an additional role in signaling at this stage in addition to leukocyte trafficking. Thus, in the eye, therapies that inhibit the production of IL-1β and activation of inflammasomes may help improve disease outcomes. An earlier study had shown in a mouse model of *P. aeruginosa* keratitis that inhibition of caspase-1 as an adjuvant therapy in combination with ciprofloxacin reduced the severity of corneal inflammation (40). Looking at our transcriptome data, we suggest that inhibition of inflammasome complex (caspase-1) could thus serves as an adjuvant therapy in targeting infectious, especially MDR retinal inflammatory diseases.

Dysregulated MMP activity has long been implicated in diseases associated with uncontrolled proteolysis of connective tissue matrices (41) along with degradation of the ECM. Therefore, it is not surprising that the retinal dissipation is higher in case of *MDR-PA* endophthalmitis along with the induced cytokine and chemokine storm and higher expression of MMPs leading to BRB breakage. The upregulated DEGs in our study included MMP-9, which was known to have elevated expression in numerous lung injury models leading to a decrease in ECM integrity (42). In addition, we observed that some intercellular tight junction proteins like claudin (9 and 7), gap junction proteins, and TGFBR2 were consistently upregulated in *MDR-P*A infection. The dysregulation of tight junctions leads to altered barrier function resulting in changes in levels of inflammatory cytokines such as IFN-α, IFN-γ, IL-6, and IL-1β as seen in inflammation-associated diseases such as multiple sclerosis and cancer (43–45). Though the role of tight and gap junction proteins are not well explored in ocular infections, a detailed study and knockout model might provide relevant information or potential targets for immunomodulation and preservation of retinal architecture. While the role of complement system is well known in the eye (46), Engelbert et al. (47) showed that its absence did not alter the disease outcome in a mice model of *Staphylococcus aureus* endophthalmitis. Interestingly, our study revealed an increased expression of some of the components of the complement system including C3a, C5a, and C5b-C9. It has been reported earlier, that in case of persistent insult and overactivation of the inflammatory response, these complements can lead to devastating tissue remodeling (48). Modulation of complement system is an active field of research in inflammation-related tissue damage (49); hence, it is critical to differentiate the beneficial roles from the detrimental roles of complement activation in MDR gram-negative endophthalmitis. Although the hyperactivation of complement system did not show any role in gram-positive bacterial endophthalmitis, its role has been well studied in H5N1 avian influenza and *Mycobacterium tuberculosis*, where it perpetrates acute lung injury and bacterial persistence by promoting the formation and maintenance of granulomas, respectively (50, 51). Also of interest is the overexpression of lipocalin (LCN-2) in our transcriptome data. This molecule is a known marker of sepsis and plays a role in attenuating bacterial growth by binding and sequestering the iron-scavenging siderophores to prevent bacterial iron acquisition. Though it has been reported that some pathogens have evolved stealth siderophores that are insensitive to LCN2 and thus can supply iron to pathogens, the present study indicates a prominent role of LCN2 in the upregulation of the cytokine storm in *MDR-PA*-infected eyes. Upregulation of LCN2 is also potentiated through MyD88-dependent NF-κB signaling following the activation of activated pattern recognition receptors (PRR), i.e., TLR4 and TLR5 (52, 53).

**Figure 8:**
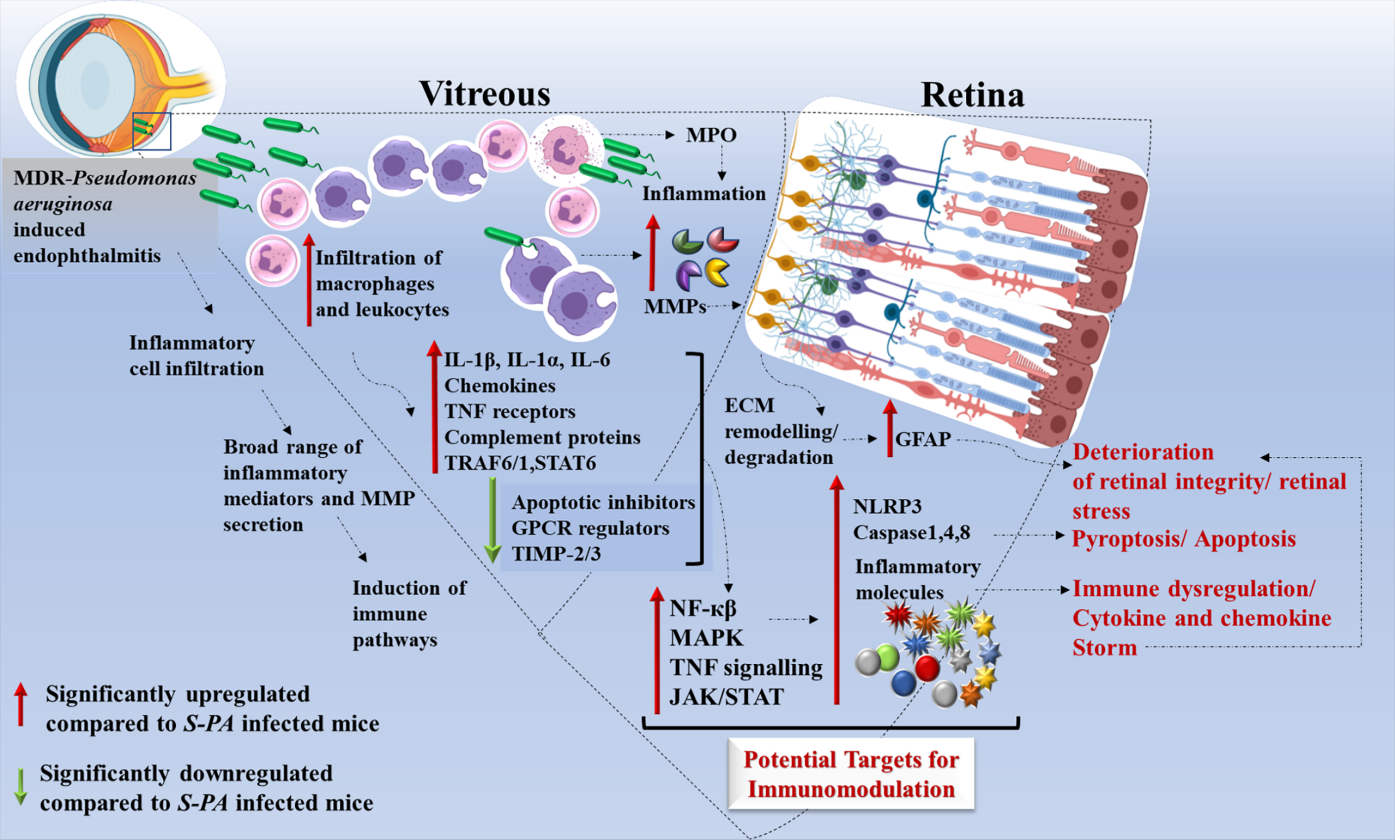
Schematic representation of host immune response induced by *multidrug-resistant Pseudomonas aeruginosa* (*MDR-PA*) in endophthalmitis. *MDR-PA* triggers an initial response which leads to higher infiltration of leukocytes and macrophages in the vitreous cavity, followed by release of cytokines (IL-1β, IL-6, IL-1α, TNF-α, IL-18), chemokines (CXCL-5, CCL-6), MPO, granzyme, and MMPs. It is beneficial for the host when the aforementioned cytokines are produced in appropriate amounts, but toxic when produced in a deregulated fashion. Transcriptome analysis revealed that pathways like NF-κβ, MAPK, TNF signaling, JAK-STAT, and complement cascade are among the top enriched pathways. IL-1β and TNF-α promote TRAF1–TRAF6–NF-κβ pathway, while IL-6 promotes STAT-6-JAK/STAT pathway which in turn can modulate the immune response. NF-κβ pathway further promotes the recruitment of immune cells and cytokines. TNF-receptor proteins are known to activate p38 and its downstream target MAPK kinase-activated kinase-2 (MK2). *MDR-PA* also activates the NLRP3 inflammasome and its main effector caspase 1, leading to the secretion of mature IL-1β and IL-18. It also activates TLR4-dependent caspase-4, leading to pyroptosis. In the presence of caspase-1, caspase-8 acts as a positive modulator of the NLRP3-dependent caspase-1 signaling cascades that drive both IL-1β production and pyroptotic death. All together, these pathways again contribute to the cytokine/chemokine storm by releasing massive amounts of proinflammatory cytokines which might be the reason behind the collateral damage. Rapid and excessive production of these molecules compared with the endophthalmitis caused by the antibiotic susceptible strain, necessitates the usage of targeted anti-inflammatory agents along with antibiotics to control and reduce the bystander damage to the retina. IL, interleukin; TNF, tumor necrosis factor; CCL, CC-chemokine ligand; CXCL, CXC-chemokine ligand; NF-κβ, nuclear factor kappa light chain enhancer of activated B cells; TRAF, tumor necrosis factor receptor–associated factor; NLRP3, NOD-, LRR-, and pyrin domain-containing protein 3.

Another important component of our comprehensive transcriptome analysis is the involvement of multiple signaling pathways and not much has been reported about its involvement in the pathogenesis of MDR infections. The highly dysregulated pathways involved were the chemokine receptor binding, TNF signaling, Toll-like receptor and NOD-like receptor signaling, NF-κB signaling, and MAP kinase signaling in *MDR-*PA-infected eyes. Chen et al. had demonstrated that in retinal ischemia and reperfusion injury, increased nuclear factor (NF)-κB p65 immunoreactivity is associated with retinal degeneration, which also seems to play an active role in MDR endophthalmitis (54). Kumar et al. (55) had demonstrated that by inhibiting NF-κB and MAP kinase signaling, inflammation along with bacterial burden was reduced in *S. aureus* endophthalmitis. Apart from the retina, studies on corneal infections have also similar findings where they have observed that *P. aeruginosa* signal the NF-κB system leading to expression and secretion of proinflammatory cytokines, IL-6 and IL-8, both of which are important in regulating PMN infiltration. In addition, the chemokine IL-1β is also a critical mediator of the innate host response to *P. aeruginosa* keratitis (56). These findings further support the involvement of NF-κB and MAP kinase in triggering an accelerated inflammatory response in *MDR-PA* endophthalmitis. While few of these genes and pathways reflect changes in metabolic requirements, most of them appear to play a role in immune evasion and pathogenesis in the eye. Altogether, our data demonstrates that *MDR-PA* infection triggers a shift toward a more immunogenic profile including an increase in inflammatory response, retinal dissipation, and aberrant pathway regulation at an earlier time point than the *S-PA* strain. A summary of our analysis is given in **Figure 8**. The major implication of our study is the suggested usage of anti-inflammatory molecules in regulating these signaling pathways to treat *MDR-PA* endophthalmitis and preserve retinal architecture and function. Our approach is novel for MDR endophthalmitis, high-impactful, translationally relevant, and will move the ocular infectious disease field forward by identifying a rational and more effective anti-inflammation strategy.

## Data Availability Statement

The original contributions presented in the study are included in the article/**Supplementary Material**, further inquiries can be directed to the corresponding author.

## Ethics Statement

The animal study was reviewed and approved by the Institutional Animal Ethics Committee (IAEC), Sipra Labs, Hyderabad, India (SLL-PCT-IAEC/20-21/B).

## Author Contributions

Conceptualization: JJ and PN. Methodology: PN, MN, DM, SB, and SP. Software: PN. Validation: SP, PN, and JJ. Resources: JJ. Writing—original draft preparation: PN. Writing—review and editing: JJ, MN, and DM. Project administration: JJ. Funding acquisition: JJ. All authors contributed to the article and approved the submitted version.

## Funding

This work was supported by the Hyderabad Eye Research Foundation (HERF) and DBT-grant (BT/PR32404/MED/30/2136/2019). We thank the ICMR SRF extrameural fellowship for the funding support to PN (OMI-fellowship/10/2020-ECD-I).

## Conflict of Interest

The authors declare that the research was conducted in the absence of any commercial or financial relationships that could be construed as a potential conflict of interest.

## Publisher’s Note

All claims expressed in this article are solely those of the authors and do not necessarily represent those of their affiliated organizations, or those of the publisher, the editors and the reviewers. Any product that may be evaluated in this article, or claim that may be made by its manufacturer, is not guaranteed or endorsed by the publisher.

## Abbreviations

BP, biological process; FDR, false-discovery rate; GO, gene ontology; KEGG, Kyoto Encyclopedia of Genes and Genomes; MF, molecular function, TIMP, tissue inhibitors of matrix metalloproteinase; NLRP3, NOD-, LRR-, and pyrin domain-containing protein 3; Cfb, complement factor B, C5ar1, complement C5a receptor 1; C1qa, complement C1q A Chain, C1qb, complement C1q B chain; C1qc, complement C1q B chain, C3, complement component 3; C4b, complement factor 4; C1ql3, complement C1q-like protein 3; JAK-STAT, janus kinase and signal transducer and activator of transcription; Rap1, repression activator protein 1; cAMP, cyclic adenosine monophosphate; Rgs7, regulator of G protein signaling 7; Gprasp2, G protein-coupled receptor-associated sorting protein 2. FADD, Fas-associated protein with death domain; BCL2L15, B-cell lymphoma 2 like 15; TRAF6, TNF receptor-associated factor 6; TRAF1, TNF receptor-associated factor 1; TNFR2, TNF receptor 2; TNFRSF1A, Tumor necrosis factor receptor superfamily member 1A.
