# Supplementary material for: Transcriptomic and Histological Analysis of Exacerbated Immune Response in Multidrug-Resistant Pseudomonas aeruginosa in a Murine Model of Endophthalmitis
Source: Front Immunol. 2022 Jan 3;12:789023. doi: 10.3389/fimmu.2021.789023 (PMC8761737; doi:10.3389/fimmu.2021.789023)
Supplement: Supplementary file 1 [file Table_1.docx]

**Supplementary Table 1**. KEGG pathways over-represented in genes that are comparatively upregulated by MDR-PA infection at 6h (FC≥ 2, FDR ≤ 0.1)

| Term | Gene Symbol | Benjanmini P value |
| --- | --- | --- |
| mmu04060: Cytokine-cytokine receptor interaction | CXCL9, IL20RB, CXCR4, CSF2RB, CXCR6, IL1RAP, IL2RG, CXCL5, CCL9, CXCR3, CCL4, CXCR2, CCL3, CCR5, CCR2, IL10RA, TNFRSF18, OSM, PPBP, TNFRSF1B, CSF2RB2, CXCL10, IL1B, IL2RB, LTB | 5.09E-07 |
| mmu04062: Chemokine signaling pathway | LYN, CXCL9, CXCR4, CXCR6, PPBP, VAV1, CXCL5, FGR, CXCL10, HCK, PLCB3, CCL9, CXCR3, CCL4, CXCR2, CCL3, RAC2, CCR5, CCR2 | 8.79E-05 |
| mmu04650: Natural killer cell mediated cytotoxicity | FCER1G, SYK, ITGB2, GZMB, VAV1, TYROBP, KLRK1, LCK, RAC2, PTPN6, LCP2, CD48, HCST | 3.31E-04 |
| mmu04145: Phagosome | NCF2, NCF4, ITGB2, CYBA, TCIRG1, CORO1A, ACTB, CTSS, FCGR1, FCGR3, CLEC7A, TLR6, H2-Q10 | 0.02 |
| mmu04666: Fc gamma R-mediated phagocytosis | FCGR1, LYN, HCK, MARCKSL1, PTPRC, SYK, RAC2, ARPC5, VAV1 | 0.02 |
| mmu04514: Cell adhesion molecules (CAMs) | CD2, SPN, VCAN, PTPRC, SELPLG, SELL, CD6, GLYCAM1, ITGB2, CD8B1, ITGB7, H2-Q10 | 0.03 |
| mmu04670: Leukocyte transendothelial migration | MYL7, NCF2, NCF4, ITGB2, RAC2, CXCR4, RHOH, CYBA, VAV1, ACTB | 0.03 |
| mmu05323: Rheumatoid arthritis | IL1B, MMP3, ITGB2, CCL3, ACP5, LTB, TCIRG1, CXCL5 | 0.06 |
| mmu05340: Primary immunodeficiency | PTPRC, LCK, CD8B1, IL2RG, CD3D | 0.1 |
| mmu04064: NF-kappa B signaling pathway | LYN, SYK, LCK, IL1B, TRADD, CCL4, LTB, BCL2A1D | 0.1 |
