# Supplementary material for: Transcriptomic and Histological Analysis of Exacerbated Immune Response in Multidrug-Resistant Pseudomonas aeruginosa in a Murine Model of Endophthalmitis
Source: Front Immunol. 2022 Jan 3;12:789023. doi: 10.3389/fimmu.2021.789023 (PMC8761737; doi:10.3389/fimmu.2021.789023)
Supplement: Supplementary file 2 [file Table_2.docx]

**Table 2**. KEGG pathways over-represented in genes that are comparatively upregulated by *MDR-PA* infection at 24h (FC≥ 2, FDR ≤ 0.1)

| Term | Genes | Benjamini |
| --- | --- | --- |
| mmu04060: Cytokine-cytokine receptor interaction | CXCL9, CSF2, CSF1, TNFRSF13B, IL24, IL1RAP, CXCL13, TNF, CXCL14, CXCL2, CXCL5, CXCL16, TNFRSF4, IFNAR2, IL11, IL15, TNFRSF18, IL1R2, TNFRSF1B, TGFBR2, CSF2RB2, IL1A, IL23A, IL1B, LTB, IFNAR1, CX3CR1, CCL12, IL4RA, CXCR4, CSF2RB, IL6RA, TNFRSF11B, IL2RG, CSF2RA, CCL9, CCL7, CXCR1, CCL6, CCL4, CXCR2, CCL3, CCL19, CCL24, GM20878, TNFSF14, IL10RB, IL10RA, OSM, IFNLR1, PPBP, INHBA, IL6, CXCL12, TNFSF9, IL7R, PF4 | 1.16E-14 |
| mmu04062: Chemokine signaling pathway | CX3CR1, CCL12, CXCL9, NCF1, WAS, CXCR4, ARRB2, CXCL3, CXCL13, CXCL2, CXCL14, CXCL5, PIK3R5, GNAI2, CXCL16, RAP1B, PREX1, CCL9, GNG2, CCL7, CXCR1, CCL6, CCL4, CXCR2, CCL3, RAC2, PTK2B, CCL19, LYN, CCL24, GM20878, PRKCD, PPBP, GNG12, NFKB1, VAV1, FGR, HCK, PLCB3, CXCL12, DOCK2, PF4, NFKBIB | 5.46E-10 |
| mmu04064:NF-kappa B signaling pathway | TNFAIP3, PTGS2, TNF, MALT1, CCL4, PLCG2, TRIM25, BCL2A1C, BCL2A1D, LBP, CCL19, CD14, LYN, SYK, TNFSF14, TRAF1, CFLAR, NFKB1, NFKB2, CXCL12, TRAF6, IL1B, BTK, LTB, TLR4, MAP3K14 | 7.49E-08 |
| mmu04666: Fc gamma R-mediated phagocytosis | LYN, VASP, MARCKSL1, SYK, NCF1, ARPC1B, PRKCD, WAS, INPPL1, ARPC5, VAV1, PIK3R5, PLD2, FCGR1, HCK, MARCKS, PTPRC, ARPC3, INPP5D, PLCG2, RAC2, DOCK2, FCGR2B | 4.45E-07 |
| mmu04670: Leukocyte transendothelial migration | ITGAM, NCF1, NCF2, ITGB2, NCF4, CXCR4, ITGAL, ACTB, PIK3R5, GNAI2, RAP1B, PLCG2, RAC2, PTK2B, VASP, ACTN2, ACTN1, MSN, RHOH, CYBA, MMP9, VAV1, CLDN11, CXCL12, CLDN9, CTNNB1 | 3.61E-06 |
| mmu04145: Phagosome | RAB7, ITGAM, NCF1, NCF2, ITGB3, H2-M3, ITGB2, H2-Q7, NCF4, TCIRG1, THBS1, CORO1A, ACTB, CTSS, C3, EEA1, CLEC7A, LAMP2, MRC1, OLR1, CD14, ITGA2, CYBA, FCGR1, MARCO, FCGR3, FCGR4, TLR6, FCGR2B, TLR4, H2-Q10, TLR2 | 5.00E-06 |
| mmu04621: NOD-like receptor signaling pathway | CCL12, CARD6, TNFAIP3, NOD1, NOD2, MEFV, TNF, CXCL2, NFKB1, NAIP2, PSTPIP1, IL6, IL1B, TRAF6, CASP1, NLRP3, NFKBIB | 9.34E-06 |
| mmu04620: Toll-like receptor signaling pathway | IFNAR2, CXCL9, CD80, FOS, TNF, NFKB1, PIK3R5, TLR1, IL6, IL1B, TRAF6, CCL4, SPP1, CCL3, MAP3K8, IRF5, LBP, TLR6, CD14, IKBKE, TLR4, IFNAR1, TLR2 | 9.34E-06 |
| mmu04668: TNF signaling pathway | CEBPB, CCL12, CSF2, CSF1, IL15, MMP3, TNFAIP3, NOD2, FOS, TRAF1, CFLAR, CXCL3, TNFRSF1B, PTGS2, TNF, MMP9, CXCL2, NFKB1, PIK3R5, IL6, IL1B, MAP3K8, MAP3K14, JUNB | 9.34E-06 |
| mmu04662: B cell receptor signaling pathway | LYN, SYK, INPPL1, DAPP1, FOS, MALT1, NFKB1, VAV1, PIK3R5, INPP5D, BTK, PLCG2, RAC2, PTPN6, PIK3AP1, FCGR2B, NFKBIE, NFKBIB | 3.37E-05 |
| mmu04144: Endocytosis | RAB7, ARPC1B, VPS4B, H2-M3, H2-Q7, WAS, CXCR4, ARRB2, CBL, IL2RG, PLD2, EEA1, RAB11FIP1, CYTH4, CXCR1, PSD4, CXCR2, KIF5A, CCDC53, LDLRAP1, EPS15, ARFGEF1, CAV3, ARAP3, ARAP1, ARPC5, IGF2R, TGFBR2, EHD1, DAB2, RAB31, ACAP1, ARPC3, TRAF6, MDM2, SMAP2, H2-Q10, HSPA1A | 1.23E-04 |
| mmu04630: Jak-STAT signaling pathway | IFNAR2, IL11, CSF2, IL15, IL10RB, IL10RA, IL24, OSM, IL4RA, IL6RA, IFNLR1, CSF2RB, IL2RG, CSF2RA, PIK3R5, CSF2RB2, IL6, IL23A, STAT4, PIM1, PTPN6, STAT6, IL7R, IFNAR1 | 9.69E-04 |
| mmu05202: Transcriptional misregulation in cancer | CEBPB, ITGAM, CSF2, CEBPE, LYL1, NFKBIZ, BCL2A1C, ITGB7, BCL2A1D, CD14, IGFBP3, IL1R2, MMP3, ETV1, TRAF1, MMP9, FLI1, NFKB1, RUNX1, TGFBR2, FCGR1, IL6, NR4A3, RARA, MDM2, REL | 0.001005216 |
| mmu04650: Natural killer cell mediated cytotoxicity | IFNAR2, FCER1G, CSF2, SYK, ITGB2, ITGAL, TNF, RAET1B, VAV1, PIK3R5, TYROBP, FCGR4, PLCG2, RAC2, PTK2B, PTPN6, LCP2, HCST, IFNAR1 | 0.001865813 |
| mmu04810: Regulation of actin cytoskeleton | CYFIP1, MYLK2, ITGAM, ITGB4, ARPC1B, ITGB3, ITGB2, WAS, IQGAP1, ITGAL, ACTB, PIK3R5, MYLK4, FGD3, RRAS, RAC2, ITGAX, ITGB7, NCKAP1L, ACTN2, ITGA2, ACTN1, FN1, MSN, ARPC5, GNG12, VAV1, ARPC3, ITGA7, ARHGEF1 | 0.001865813 |
| mmu04510: Focal adhesion | MYLK2, ITGB4, ITGB3, LAMA3, THBS1, ACTB, PIK3R5, MYLK4, RAP1B, SPP1, RAC2, FLNA, FLNB, ITGB7, FLNC, VASP, LAMB3, CAV3, LAMB2, ACTN2, ITGA2, ACTN1, FN1, VAV1, VEGFA, ZYX, ITGA7, CTNNB1, TLN1 | 0.002577163 |
| mmu05321: Inflammatory bowel disease (IBD) | IL4RA, NOD2, IL2RG, TNF, NFKB1, IL1A, IL6, IL23A, IL1B, STAT4, STAT6, TLR4, TLR2 | 0.003846471 |
| mmu04610: Complement and coagulation cascades | C1QB, C1QA, F10, SERPIND1, C5AR1, PLAUR, F13A1, F3, C3, C4B, THBD, CD55, CFB, C1QC | 0.011133952 |
| mmu04664: Fc epsilon RI signaling pathway | LYN, FCER1G, CSF2, SYK, PLA2G4A, TNF, VAV1, PIK3R5, INPP5D, BTK, PLCG2, RAC2, LCP2 | 0.012324013 |
| mmu04010: MAPK signaling pathway | HSPB1, ARRB2, DUSP16, TNF, RAP1B, RRAS, RAC2, FLNA, FLNB, MAP3K8, CD14, FLNC, DUSP2, GADD45B, GADD45A, DUSP1, PLA2G4A, FOS, GNG12, NFKB1, GADD45G, NFKB2, TGFBR2, NR4A1, IL1A, TRAF6, IL1B, MAPKAPK2, MAP3K14, HSPA1A | 0.019376281 |
| mmu04660: T cell receptor signaling pathway | CSF2, FOS, TNF, CD3D, MALT1, NFKB1, VAV1, PIK3R5, PTPRC, PTPN6, MAP3K8, LCP2, NFKBIE, MAP3K14, NFKBIB | 0.043571575 |
| mmu04142: Lysosome | NAPSA, SLC11A1, CTSZ, LAPTM5, TCIRG1, LITAF, GNS, IGF2R, CTSS, CLN5, CLN3, SMPD1, LAMP2, ACP5, CTSH, MAN2B1, CD68 | 0.043571575 |
| mmu04520: Adherens junction | ACTN2, ACTN1, SNAI1, WAS, RAC2, CTNNB1, PTPN6, LMO7, IQGAP1, SORBS1, ACTB, TGFBR2 | 0.046726949 |
| mmu04512: ECM-receptor interaction | LAMB3, ITGB4, LAMB2, ITGB3, ITGA2, LAMA3, FN1, THBS1, HSPG2, SPP1, ITGA7, ITGB7, CD47 | 0.077981955 |
| mmu05100: Bacterial invasion of epithelial cells | CAV3, ARPC3, ARPC1B, RHOG, WAS, FN1, CTNNB1, HCLS1, ARPC5, CBL, ACTB, PIK3R5 | 0.078350417 |
| mmu04066: HIF-1 signaling pathway | PFKFB3, NOS2, CAMK2A, SLC2A1, IL6RA, ENO1, HIF1A, NFKB1, VEGFA, PIK3R5, HK3, IL6, PLCG2, TLR4 | 0.098053124 |
| mmu04151: PI3K-Akt signaling pathway | CSF1, ITGB4, ITGB3, LAMA3, IL4RA, IL6RA, IL2RG, THBS1, PIK3R5, GYS1, BCL2L11, GNG2, SPP1, ITGB7, MCL1, IFNAR2, LAMB3, SYK, LAMB2, ITGA2, OSM, FN1, GNG12, NFKB1, VEGFA, NR4A1, IL6, MDM2, ITGA7, IL7R, PIK3AP1, TLR4, EPHA2, TLR2, IFNAR1 | 0.099228026 |
| mmu05200: Pathways in cancer | PTGER4, LAMA3, SLC2A1, CXCR4, PTGS2, CBL, HIF1A, CSF2RA, PIK3R5, GNAI2, GNG2, PLCG2, RAC2, E2F2, LAMB3, NOS2, LAMB2, ITGA2, FN1, FOS, TRAF1, GNG12, MMP9, NFKB1, VEGFA, RUNX1, NFKB2, TGFBR2, IL6, PLCB3, CXCL12, TRAF6, RARA, CKS2, MDM2, CTNNB1, ARHGEF1, BIRC7 | 0.112626578 |
| mmu04530: Tight junction | CLDN11, MYH1, MYH2, MYH3, ACTN2, CLDN9, ACTN1, MYH13, HCLS1, MYH9, AMOTL1, ACTB | 0.172253112 |
